# A unified vision-language model for cross-product defect detection in glove manufacturing

**DOI:** 10.1371/journal.pone.0339867

**Published:** 2026-02-11

**Authors:** Yusen Zhao, Liang Tian, Yonggang Wang

**Affiliations:** 1 Institute of Applied Mathematics, Hebei Academy of Sciences, Shijiazhuang, Hebei, China; 2 Hebei Province Information Security Certification Technology Innovation Center, Shijiazhuang, Hebei, China; 3 School of Mathematical Sciences, Hebei Normal University, Shijiazhuang, Hebei, China; 4 Hebei Digital Education Collaborative Innovation Center, Shijiazhuang, Hebei, China; Dalian Maritime University, CHINA

## Abstract

Automated anomaly detection is vital to industrial quality control, yet conventional deep learning detectors often struggle with scalability. These models, typically following a rigid “one-model-per-task” paradigm, require separate systems for each product line, increasing operational complexity and cost in diverse manufacturing environments. To address this limitation, we propose a unified defect detection framework based on a Multimodal Large Language Model (MLLM). Our approach utilizes a two-stage fine-tuning strategy: Supervised Fine-Tuning (SFT) to impart domain-specific knowledge, followed by a novel Reinforcement Fine-Tuning (RFT) process that refines visual reasoning. This RFT stage is guided by a multi-faceted verifiable reward function designed to optimize localization accuracy, classification correctness, and output structure. On a challenging real-world glove manufacturing dataset, our RFT-enhanced MLLM achieves a mean Average Precision (mAP) of 0.63, which is comparable to a highly specialized YOLO baseline (0.62). More importantly, a single, unified MLLM trained on a mixed-product dataset maintains competitive performance (mAP 0.61), demonstrating its ability to dynamically handle different products and defect types via natural language prompts. This study validates the feasibility of using a single, flexible MLLM to replace multiple rigid models in complex industrial inspection, offering a scalable and cost-effective paradigm for future intelligent quality control systems. The open-source code will be released at https://github.com/GloamXun/Glove-MLLM.

## Introduction

Nitrile rubber gloves are a critical component in personal protective equipment, with demand growing exponentially across medical and industrial applications. The intricate manufacturing process, however, presents persistent quality control challenges where even minor defects can lead to significant safety risks. While manual inspection is common, it is inconsistent and inefficient, necessitating automated anomaly detection systems. However, real-world production environments pose a confluence of dynamic challenges that degrade model performance. The persistent vibration of machinery causes irregular poses like folds and twists, which are often misclassified by conventional models. This complexity is compounded by variations in lighting and backgrounds, which introduce significant noise and make robust detection difficult. Addressing this multitude of challenges—dynamic poses, environmental shifts, and defect heterogeneity—is a key barrier to deploying effective automated systems.

In a typical deployment scenario using conventional detectors like YOLO [1], this diversity necessitates training, deploying, and maintaining a separate, specialized model for each distinct product type. This approach, while effective for a single task, quickly becomes cumbersome and costly as the product line expands, increasing the total cost of ownership and operational complexity.

Furthermore, the challenge is not merely visual; different products often require monitoring for entirely different sets of defect classes, dictated by their material properties and manufacturing processes. For instance, based on the specific quality control priorities of our partner factory, defects like material adhesion are deemed critical for blue nitrile gloves, while for white PVC gloves, the inspection focus shifts to issues such as oil stains and cuff rolling defects. This means that even if a defect type could technically occur on multiple products, the inspection protocol prioritizes it differently depending on the product line. Handling this with a conventional approach would demand not just separate models, but distinct annotation schemas and training pipelines for each product-class combination, further compounding the problem.

To address this core issue of scalability and flexibility, we hypothesize that a single Multimodal Large Language Model (MLLM), guided by natural language instructions, can serve as a unified inspection system. Such a model could overcome the limitations of the “one model per product” paradigm by dynamically adapting to different products and their specific defect requirements. This study, therefore, aims to build and evaluate such a system, providing a direct comparative analysis against a specialized YOLO baseline to highlight the trade-offs between targeted accuracy and unified operational efficiency.

In summary, this research makes the following key contributions:

- We introduce and benchmark on a complex, realistic industrial dataset featuring mixed products (blue nitrile and white PVC gloves) with product-specific defect classes, simulating a challenging real-world quality control scenario.
- We demonstrate the semantic flexibility of a single, unified MLLM that, guided by natural language prompts, achieves detection accuracy comparable to a specialized YOLO baseline while dynamically handling multiple, distinct product lines.
- We provide a comparative analysis of the “one-model-per-task” versus the “unified-model” paradigm, concluding that for multi-product environments, the MLLM’s operational flexibility offers a significant advantage in cost-effectiveness and scalability not captured by accuracy metrics alone.

## Related work

### Automated defect detection in manufacturing

Defect classification and detection are critical for quality control in industrial manufacturing. Traditional manual inspection, while prevalent, suffers from inefficiency and subjective variability, rendering it inadequate for modern industrial demands. Early automated approaches relied on traditional image processing techniques; for instance, Gaidhane et al. [2] explored edge detection and template matching, which performed well in controlled scenarios but faltered in complex environments. The integration of statistical learning methods, such as support vector machines (SVMs) utilized by Krummenacher et al. [3] and random forests applied by Shipway et al. [4], improved robustness but still relied on hand-crafted feature extraction.

The advent of deep learning, particularly Convolutional Neural Networks (CNNs), revolutionized the field, as surveyed by Jha [5] and Mezher [6]. In the specific domain of glove inspection, recent innovations have demonstrated remarkable performance. For example, Jin et al. [7] developed CCA-YOLO, an enhanced variant of the YOLOv5 architecture originally proposed by Jocher et al. [8], which achieved mean Average Precision (mAP) scores exceeding 99% on certain defect categories by addressing challenges specific to thin-film elastomers. However, despite their high accuracy, these CNN-based detectors are inherently specialized. They operate under a rigid “one-model-per-task” paradigm, lacking the semantic flexibility to handle diverse product lines or adapt to new defect types without complete retraining. This fundamental limitation has motivated the exploration of more flexible, language-driven models. Some recent studies have begun to train MLLMs specifically for industrial anomaly detection, such as AnomalyGPT by Gu et al. [9], Myriad by Li et al. [10], and FabGPT by Jiang et al. [11]. While promising, these approaches often rely on existing expert models and cannot freely extend their capabilities by changing inputs, leaving a gap for a truly unified, cross-product solution.

### Multimodal large language models for visual reasoning

Recent advancements in Multimodal Large Language Models (MLLMs) have significantly enhanced the ability of AI systems to process and reason about visual information. Pioneering studies, such as the QVQ series by the Qwen Team [12] have proposed vision-based reasoning frameworks that expand MLLMs’ capabilities in interpreting complex visual data, laying the groundwork for adapting these models to specialized tasks like defect detection. Typically, MLLM training follows a two-stage paradigm: large-scale pre-training on multimodal corpora, followed by post-training involving Supervised Fine-Tuning (SFT) for instruction following. This process endows MLLMs with the ability to perform complex visual reasoning tasks based on natural language prompts. Unlike conventional detectors that output only bounding boxes and class labels, MLLMs can generate rich, interpretable textual justifications for their predictions, offering a new level of transparency. However, adapting these general-purpose models to highly specific, structured-output tasks like industrial defect detection remains a significant challenge. This performance gap has been systematically quantified by recent comprehensive benchmarks. For instance, the MMAD benchmark presented by Jiang et al. [13] revealed that even state-of-the-art models like GPT-4o fall far short of industrial accuracy requirements, highlighting a critical need for domain-specific adaptation and fine-tuning.

### Reinforcement learning for MLLM alignment

To bridge the gap between general visual reasoning and specialized task performance, Reinforcement Learning (RL) has emerged as a powerful fine-tuning technique. The success of models like OpenAI’s o1 [14] demonstrates that RL can significantly enhance reasoning capabilities beyond what SFT alone can achieve. Most existing RL approaches for MLLMs utilize Proximal Policy Optimization (PPO), an algorithm introduced by Schulman et al. [15], a policy gradient algorithm ensuring stable training. Innovating beyond this, Shao et al. [16] have proposed methods like Group Relative Policy Optimization (GRPO), which eliminates the need for a critic model and improves computational efficiency.

While RL has been extensively applied for mitigating hallucinations as shown by Sun et al. [17] and aligning with human preferences as explored by Yu et al. [18], its potential to enhance domain-specific visual perception is largely underexplored. The Visual-RFT framework proposed by Liu et al. [19] begins to address this gap by introducing verifiable reward functions to advance MLLM capabilities in classification and detection. Our work builds directly upon this foundation. We adapt the RFT paradigm to the industrial domain by designing a novel, multi-faceted verifiable reward function tailored specifically for cross-product defect detection, establishing a methodological framework for applying advanced MLLM alignment techniques to complex, real-world manufacturing challenges.

## Materials and methods

### Data preparation

To evaluate our approach, we constructed a challenging dataset designed to mirror real-world industrial complexities. Our data collection was conducted in cooperation with a medical glove factory across several production lines. For this purpose, a specialized, visual inspection system, custom-designed by an automation partner, was deployed on each line. While this system is proprietary, its configuration was standardized across all lines to ensure consistent image acquisition.

The system features a six-camera array. Each production line consists of two parallel lanes for glove manufacturing, with three cameras assigned to each lane. The cameras are strategically positioned: two capture the side-views of the glove (covering the palm and the back), while a third camera captures the bottom view. This study primarily utilizes the images obtained from the side-view cameras, as they are effective for identifying most target defects.

The dataset is composed of two primary subsets:

- **Blue Nitrile Glove Subset:** This forms the foundation of our dataset, comprising 23,799 images with 27,083 annotated bounding boxes. The primary defects of concern for this product are adhesion, tearing, and contamination.
- **White PVC Glove Subset:** To test generalization, we extended the dataset with over 11,378 images of white PVC gloves. For this product, the inspection focus shifts to oil stains (a specific contamination type), tearing, and Cuff Rolling Defect, a unique flaw where the glove’s cuff is improperly rolled.

A detailed understanding of these defect types is crucial. The defects we focus on include adhesion, tearing, contamination, and cuff rolling. Adhesion defects occur when raw materials stick to the glove mold, often caused by mold surface imperfections (e.g., cracks or damage). This can lead to material thinning and structural weakness, predisposing the gloves to tearing. Tearing defects manifest as ruptures, typically during demolding, resulting from excessive mechanical stress. Contamination involves foreign substances like dust particles or oil stains. The cuff rolling defect refers to an improperly rolled cuff at the wrist-end, which can compromise the glove’s fit and barrier function.

**Fig 1:**
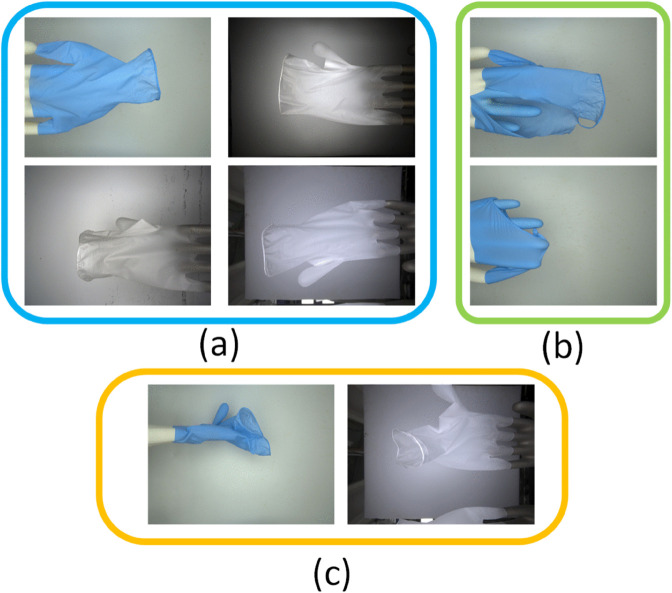
Illustration of complex challenges in real-world industrial inspection. (a) Four images demonstrating variations in lighting conditions due to different equipment deployment environments and complex backgrounds like stained backdrops. (b) Examples of various types of damage and tearing defects. (c) Two images showing unstable glove poses during capture, caused by the shaking of glove molds.

A crucial characteristic of this dataset is the presence of challenges mirroring real-world production environments, as illustrated in Fig 1. The persistent vibration of machinery causes gloves on the production line to shake, leading to irregular poses such as folds and twists during image capture. These motion-induced deformations create visual artifacts like creases that are often misclassified as tearing defects by conventional models. The complexity is further compounded by environmental and operational variations. Different production lines may have inconsistent lighting conditions, and over time, the inspection background can accumulate dust or stains. These factors introduce significant noise and variability into the captured images, making it difficult to maintain robust detection accuracy.

Another characteristic of this dataset is the presence of inherent label noise. During the manual annotation process, we observed that the precise boundaries and definitions of certain defects were subject to the varying interpretations of different annotators. This lack of a single, rigid standard for bounding box placement introduces a level of ambiguity that reflects real-world conditions and makes achieving exceptionally high mAP scores challenging for any model. This “noisy” but realistic dataset serves as a robust testbed for evaluating model performance under practical constraints.

### Fine-tuning strategy

Our approach is centered on a two-stage fine-tuning strategy to adapt a MLLM for the unified inspection task. The first stage, Supervised Fine-Tuning (SFT), serves to inject essential domain-specific knowledge, teaching the model the basic visual characteristics of glove defects and the required output format. The second, more critical stage is Reinforcement Fine-Tuning (RFT), which moves beyond simple pattern matching to refine the model’s visual reasoning and localization abilities. This stage is designed to enhance the model’s robustness in handling the complex and ambiguous scenarios prevalent in real-world industrial environments.

To implement the RFT stage, we employ Group Relative Policy Optimization (GRPO) [16], a policy gradient algorithm. GRPO operates by sampling a group of candidate responses from the model’s current policy and evaluating them using a reward signal. It then normalizes these rewards to form an advantage estimate, which guides the policy update. We selected GRPO for its computational efficiency, as it eliminates the need for a separate critic model required by other algorithms like PPO. In our framework, the crucial reward signal is provided not by a trained reward model, but by a rule-based, verifiable reward function, which is detailed in the following section.

### Verifiable reward function

To align the RFT process with the defect detection task, we replace a generic reward model with a verifiable, task-specific reward function. In detection, the primary criteria are generating precise bounding boxes and classifying objects correctly. This allows for the construction of rule-based rewards aligned with performance metrics. Our novel verifiable reward function comprises four key components: a CIoU reward for localization, a category reward for classification, a confidence reward for calibration, and a format reward for structural integrity.

#### CIoU reward.

In object detection tasks, Intersection over Union (IoU) is a fundamental metric for evaluating bounding box regression accuracy. It quantifies the overlap ratio between the predicted box *B*_*pred*_ and the ground truth box *B*_*gt*_. While IoU is intuitive for measuring overlap, it suffers from critical limitations as a comprehensive evaluation criterion. IoU exhibits geometric ambiguity by assigning identical scores to boxes with divergent spatial configurations (e.g., misaligned centers or mismatched aspect ratios), thereby failing to capture positional precision and shape consistency required for industrial defect localization. To address these shortcomings, Complete IoU (CIoU) [20] extends the IoU metric by integrating geometric completeness:

$$\text{CIoU}=IoU-\frac{\rho^{2}(b_{bred},b_{gt})}{c^{2}}-\beta v$$ (1)

where $\rho^{2}(b_{pred},b_{gt})$ penalizes the Euclidean distance between box centers. *c* is the diagonal length of the minimum enclosing rectangle. *v* quantifies aspect ratio discrepancy, and it’s calculated as follow:

$$v=\frac{4}{\pi^{2}}{\left(\arctan\frac{w_{gt}}{h_{gt}}-\arctan\frac{w_{pred}}{h_{pred}}\right)}^{2}$$ (2)

and $\beta =\frac{v}{(1-\text{IoU})+v}$ balances the contribution of shape mismatch.

For the model, we expect the model giving a group of coordinates of the bounding boxes and we evaluate the CIoU between output bounding boxes $\left\{b_{pred1},b_{pred_{2}},...,b_{pred_{G}}\right\}$ and ground truth *b*_*gt*_. For the *i*-th output in this group which contains *n* predicted bounding boxes, the CIoU reward would be like:

$$R_{CIoU}^{i}=\frac{\sum_{k=1}^{n}ciou_{k}}{n}$$ (3)

As mathematically derived, when two bounding boxes are perfectly aligned, the IoU metric in Equation 1 achieves its upper bound of 1 with both penalty terms reducing to exactly 0, thereby establishing the theoretical maximum value of CIoU. Conversely, for non-overlapping bounding boxes, the IoU component collapses to 0 while the geometric penalty terms drive the CIoU value below zero. This formulation creates a self-regularizing mechanism: accurate predictions yield positive $R_{\text{CIoU}}$ values that reinforce correct localization, whereas erroneous bounding boxes generate negative reward signals ($R_{\text{CIoU}}<0$) that transform the reward into a penalty. Such bidirectional feedback enables the model to dynamically distinguish between valid and invalid predictions during optimization.

#### Confidence reward.

The confidence reward is designed to encourage the model to assign a high score to correct bounding boxes. Specifically, the confidence reward is related to the CIoU metric. For the *k*-th bounding box in output $b_{{\text{pred}}_{i}}$, the confidence reward is defined as:

$$r_{conf}^{k}=\left\{ \begin{array}{cc} conf_{k} & CIoU>0\\ 1-conf_{k} & CIoU\leq 0 \end{array}\right.$$ (4)

Equation 4 indicates that if the predicted bounding box is accurate, the model should assign a higher confidence score. Conversely, it should assign a lower confidence score for poor predictions. The overall confidence reward is computed as:

$$R_{conf}^{i}=\frac{\sum_{k=1}^{n}r_{conf}^{k}}{n}$$ (5)

#### Category reward.

The category reward evaluates whether the model provides correct defect classifications. It assigns a reward of 1 for correct classifications and 0 otherwise. Formally, the category reward is defined as:

$$R_{cat}^{i}=\frac{\sum_{k=1}^{n}1_{\left\{o_{k}==gt\right\}}}{n}$$ (6)

where *o*_*k*_ denotes the model’s *k*-th classification output, and *gt* represents the corresponding ground truth label. The indicator function $1_{\left\{o_{k}==gt_{k}\right\}}$ returns 1 when the prediction matches the ground truth, and 0 otherwise. For the *i*-th output group, the category reward is the mean accuracy over all predictions.

Additionally, in the defects detection task, we may provide a qualified glove image which has no defects on it. As our rules, the model should output ‘No Defects’ on this type of image. So if the model correctly identify the image as ‘No Defects’, we reward 1 as well.

#### Format reward.

The final component of our verifiable reward function is the format reward. We employ RFT to endow the MLLM with reasoning capabilities. To enforce structured thinking, we first incorporate format specifications in the user prompt (see Table 1), inspired by the user prompt design in Visual-RFT [19]. The second part of the table demonstrates the required output format: the model should encapsulate its reasoning process between <think> and </think> tags, with the final answer enclosed within <answer> and </answer> tags. And for RFT prompt, we need to adjust the specific defect class names to fit different production. However, given the hallucination tendencies of large language models, strict format compliance cannot be guaranteed. We therefore introduce format rewards to incentivize proper output structuring.

**Table 1:** The format of SFT and RFT dataset.

Algorithm	Content	
2*SFT	**Question**	<image> Detect all defects in the image, and provide the bounding boxes (between 0 and 1000, integer) and confidence (between 0 and 1, with two decimal places) and category (string). If no defect belonging to the category mentioned above in the image, return ‘No Defects’. The output answer format should be as follows: [‘Position’: [x1, y1, x2, y2], ‘Confidence’: number, ‘Category’: string, ...]. Please strictly follow the format.
	**Solution**	[‘Position’: [413, 700, 483, 725], ‘Confidence’: 1.0, ‘Category’: ‘contamination’]
2*RFT	**Question**	Detect all defects belonging to the category {defects classes} in the image, and provide the bounding boxes (between 0 and 1000, integer) and confidence (between 0 and 1, with two decimal places) and category (string). If no defect belonging to the category mentioned above in the image, return ‘No Defects’. Output the thinking process in < think> < /think> and final answer in < answer> </answer> tags.The output answer format should be as follows: < think>... </think> < answer>[‘Position’: [x1, y1, x2, y2], ‘Confidence’: number, ‘Category’: string, ...]</answer>. Please strictly follow the format.
	**Solution**	< answer>[‘Position’: [759, 583, 930, 961], ‘Confidence’: 1.0, ‘Category’: ‘tear’]</answer>

This table illustrates the data format for SFT and a specific format for RFT prompt. The ‘Question’ column shows the prompt template, and the ‘Solution’ column provides an example of the expected output. The ‘{defects classes}’ part should be specific to each dataset.

The format reward comprises two components. First, we verify the presence of required tags: <think>, </think>, <answer>, and </answer>. A base reward of 0.5 ($r_{\text{tag}}$) is awarded for correct tag usage. The second component evaluates the reasoning length to prevent gaming of the reward system. Without length constraints, models might simply repeat instructions within the tags to bypass actual reasoning. We therefore stipulate that the reasoning process must contain more than 50 words but fewer than 250 words , awarding an additional 0.5 ($r_{\text{len}}$) for compliance. This length constraint encourages concise yet sufficient reasoning, preventing both overly simplistic responses and verbose, uninformative outputs that might arise from reward hacking. The composite format reward for the *i*-th output group is defined as:

$$R_{format}^{i}=\frac{\sum_{k=1}^{n}\left(r_{\text{tag}}^{k}+r_{\text{len}}^{k}\right)}{n}$$ (7)

#### Composite reward formulation.

The verifiable reward function integrates the four component rewards established in previous sections through a summation scheme. In GRPO algorithm, we generate *G* candidate predictions $\left\{b_{{\text{pred}}_{1}},b_{{\text{pred}}_{2}},\ldots ,b_{{\text{pred}}_{G}}\right\}$ through strategic sampling. For the *i*-th output, the composite reward *R^i^* is formulated as:

$$R^{i}=\underset{\text{Localization}}{\underbrace{R_{\text{CIoU}}^{i}}}+\underset{\text{Confidence}}{\underbrace{R_{\text{conf}}^{i}}}+\underset{\text{Category}}{\underbrace{R_{\text{cat}}^{i}}}+\underset{\text{Format}}{\underbrace{R_{\text{format}}^{i}}}$$ (8)

This multi-faceted reward structure is designed to provide a comprehensive and balanced learning signal. Each component addresses a distinct and relatively orthogonal aspect of the task: The CIoU reward *R*_*CIoU*_ focuses exclusively on spatial accuracy, guiding the model to generate precise bounding boxes. The category reward *R*_*cat*_ targets semantic correctness, ensuring the defect is correctly classified. The confidence reward *R*_*conf*_ addresses model calibration, encouraging the model to align its predicted confidence with localization quality. The format reward *R*_*format*_ enforces structural integrity, promoting clear, interpretable, and parsable outputs.

While a weighted sum of these components could be explored, we opted for a simple summation for its robustness and simplicity. A key reason this approach is effective lies in the nature of the GRPO algorithm. GRPO does not use the raw reward values directly. Instead, it normalizes the rewards within a sampled group to compute advantage estimates ($A_{i}=\frac{r_{i}-mean(r)}{std(r)}$). This normalization process inherently mitigates the issue of varying scales among different reward components. Consequently, the optimization is driven by the relative ranking of candidate responses within a group, rather than the absolute magnitude of the rewards. This allows the model to learn to simultaneously improve on all four fronts without one component’s scale dominating the learning signal. While an exhaustive ablation study could further refine the contribution of each component, our current design proved empirically effective, leading to significant performance gains over the SFT baseline as shown in Table 2. This holistic guidance is critical for adapting MLLMs to a structured-output task like object detection.

**Table 2:** Comparison of mAP and Precision across different models.

Model	Training Method	mAP	Precision
YOLO11-L	Supervised	0.62	0.90
Qwen2-VL-2B	SFT-only	0.53	0.85
**Qwen2-VL-2B**	**SFT+RFT(Ours)**	**0.58**	**0.89**
Qwen2.5-VL-3B	SFT-only	0.56	0.87
**Qwen2.5-VL-3B**	**SFT+RFT(Ours)**	**0.63**	**0.92**

A comparison of performance metrics for different models and training strategies. Bolded rows indicate results from our proposed SFT+RFT method.

## Experimental and results

### Models and baselines

To balance computational efficiency and practical deployment feasibility, we employ 3B-parameter models as our experimental framework, specifically selecting the Qwen2-VL-2B [21] and Qwen2.5-VL-3B [22] as baseline references. These models are chosen for their optimal trade-off between inference speed, hardware compatibility, and multimodal processing capabilities. For systematic comparison, SFT is implemented on both models using the complete training dataset, while the YOLO11-L model [23] – trained under identical data conditions – serves as the baseline for evaluating defect detection metrics.

### Implementation details

During experiments, all images are resized to 1024×768 pixels to balance computational and memory constraints. Prior to initiating RFT, we inject domain-specific prior knowledge into the base model through SFT, as the original model lacks expertise in nitrile or PVC glove defect recognition. This preliminary SFT phase utilizes the RFT dataset, with corresponding training prompts detailed in the SFT section of Table 1. The models undergo 10-epoch training on this subset to establish foundational defect detection capabilities. Following SFT completion, the optimized SFT-models proceed to the RFT stage, which executes over 2 epochs with configuration parameters set to a maximum image resolution of 802,816 pixels and a context length limit of 4,096 tokens.

### Evaluation metrics

The primary metric used to evaluate model performance is mean Average Precision (mAP). mAP provides a comprehensive assessment of an object detector’s performance by considering both Precision (the accuracy of predictions) and Recall (the model’s ability to find all ground-truth objects). All models are evaluated on a held-out test set to ensure fair comparison.

### Results

#### Experiment 1: Ablation study on a single-product dataset.

The objective of this first experiment was to validate our RFT methodology and establish a performance baseline on a standard task. For this purpose, we used only the Blue Nitrile Glove Subset. The results are presented in Table 2. We use Mean Average Precision (mAP) as the primary metric to evaluate our models. mAP provides a comprehensive assessment of an object detector’s performance. The calculation of mAP is based on two fundamental metrics: Precision, which measures the accuracy of the predictions (i.e., what proportion of predicted boxes are correct), and Recall, which measures the model’s ability to find all ground-truth objects.

As shown in Table 2, our RFT-enhanced MLLM, specifically the Qwen2.5-VL-3B SFT+RFT(Ours) model, achieves an mAP of 0.63. This score is on par with, and slightly exceeds, the highly-optimized YOLO11-L baseline (mAP0.62). The modest absolute mAP values across all models can be attributed to the previously mentioned label noise in the dataset, which makes high-precision localization inherently difficult.

A key finding from our ablation study is the substantial performance uplift provided by RFT over SFT. For instance, the Qwen2.5-VL-3B model, when enhanced with RFT, sees its mAP jump from 0.56 (SFT-only) to 0.63. A similar trend is observed with the Qwen2-VL-2B model, which improves from 0.53 to 0.58 mAP. This demonstrates that while SFT provides a solid foundation for domain knowledge, the RFT stage is critical for refining the model’s reasoning and localization abilities. The verifiable reward function, which directly optimizes for CIoU, classification accuracy, and confidence, forces the model to generate outputs that are not just plausible but verifiably correct—a level of precision that standard cross-entropy loss in SFT struggles to achieve for such structured output tasks.

**Fig 2:**
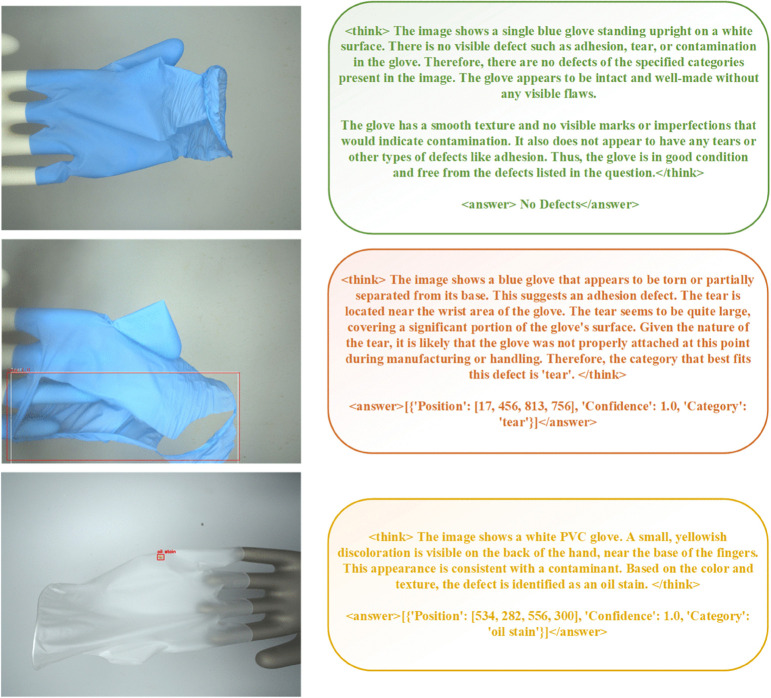
This figure demonstrates the model’s inference results on three distinct samples: a normal blue nitrile glove, a torn blue nitrile glove, and a white PVC glove with an oil stain. The first case highlights the model’s robustness; its ability to correctly identify the rolled cuff of the normal glove, a feature often misclassified as a tear by other models, shows superior handling of gloves with special morphologies.

Furthermore, we observed qualitatively that the RFT-enhanced models produced more reliable and interpretable reasoning chains within the <think> tags. As illustrated by the examples in Fig 2, the model demonstrates a nuanced understanding of the task. For instance, it correctly identifies a normal glove’s rolled cuff, a feature often misclassified as a ‘tear’ by purely vision-based models that may overfit to specific edge patterns. We hypothesize that this improved robustness stems from the RFT process, which forces the model to generate not just a bounding box, but also a coherent textual justification. By rewarding the entire reasoning process, the model learns to base its decisions on a more holistic understanding of the visual evidence, rather than relying on superficial or potentially misleading features. This capability to handle challenging cases with visual ambiguity, which were common failure points for the SFT-only and YOLO models in our observations, underscores the potential of MLLMs to bring a new level of reasoning to industrial inspection tasks.

#### Experiment 2: Evaluating semantic flexibility on the mixed-product dataset.

Having established the efficacy of our RFT method, this second experiment was designed to directly compare the training flexibility of MLLMs against the rigid nature of conventional detectors in a multi-product setting. We aimed to demonstrate that while both models can be trained as specialists, only the MLLM can function as a unified generalist.

To achieve this, we conducted a series of training runs:

- Specialist Models: YOLO11-L and Qwen2.5-VL-3B were each trained separately on the Blue Nitrile Glove subset and the White PVC Glove subset.
- Unified Generalist Model: The Qwen2.5-VL-3B was also trained on the full, combined dataset of both blue and white gloves, with their distinct defect schemas, to test its ability to function as a single, unified system.

The results, which highlight the different operational paradigms, are presented in Table 3.

**Table 3:** Comparison of mAP and Precision across different models.

Model	Training Dataset	Training Schema	mAP	Precision
YOLO11-L	Blue Nitrile Only	Nitrile Defects	0.62	0.90
YOLO11-L	White PVC Only	PVC Defects	0.64	0.91
Qwen2.5-VL-3B	Blue Nitrile Only	Nitrile Defects	0.63	0.92
Qwen2.5-VL-3B	White PVC Only	PVC Defects	0.65	0.93
Qwen2.5-VL-3B	Mixed (Blue+White)	Unified Schema	0.61	0.90

This table compares the performance of YOLO11-L and Qwen2.5-VL-3B models when trained on different datasets and schemas.

The results in Table 3 clearly illustrate the core trade-offs. When trained as specialists on a single product type, both YOLO and our MLLM perform exceptionally well, with the MLLM even achieving a slightly higher peak performance to 0.65 mAP on the white PVC dataset. This confirms that MLLMs are highly competent detectors for standard, single-task problems.

However, the most significant result is found in the last row of the table. The unified MLLM, trained on the mixed dataset, achieves a competitive mAP of 0.61. While this represents a minor performance trade-off compared to its specialist counterparts, its accomplishment is profound: this single model performs the work of two separate, specialized YOLO models. It dynamically processes images of different products and applies the correct, distinct defect detection rules as instructed by the prompt.

This experiment validates our central hypothesis. The conventional YOLO approach is confined to a “one model per task” pipeline. To handle our scenario, a factory would need to deploy and maintain two distinct YOLO models with separate training pipelines, deployment configurations, and maintenance schedules. In stark contrast, our approach enables a single, unified MLLM to cover the entire scope. As illustrated by the sample outputs in Fig 2, the MLLM not only overcomes detection challenges posed by special glove morphologies (as seen in the first sample) but also provides a concise and intuitive rationale to justify each finding. This inherent flexibility to be trained on mixed data with heterogeneous tasks represents a paradigm shift, offering a dramatic reduction in operational complexity and total cost of ownership, thereby proving its superior value for diverse, real-world industrial environments.

## Discussion and conclusion

This study demonstrates a significant advancement in automated anomaly detection by validating a unified, language-driven MLLM for cross-product defect detection. Our principal finding is that a single MLLM, enhanced by a two-stage fine-tuning process, can achieve detection accuracy (mAP of 0.63) comparable to a highly specialized YOLO baseline (mAP of 0.62) on a single-product task. More importantly, this unified model maintains competitive performance (mAP of 0.61) on a mixed-product dataset, a task that conventional detectors cannot perform with a single model. This confirms that MLLMs offer a viable “one-model-for-all” paradigm, drastically reducing the operational complexity and cost associated with deploying and maintaining multiple task-specific models in diverse manufacturing environments.

The practical application of this paradigm, however, involves a trade-off between flexibility and computational efficiency. The inherent latency of autoregressive MLLMs currently makes our model better suited for applications where adaptability is paramount, such as offline quality auditing or process development. Furthermore, complex glove deformations in real-world settings present a dual challenge for any inspection system. The first challenge is occlusion, where a fold may obscure a genuine defect, a fundamental limitation for vision-based methods that can lead to false negatives. The second, more subtle challenge is visual ambiguity, where benign features of a deformed glove—such as folds or a rolled cuff—are misidentified as defects by conventional models. This latter issue is a primary cause of high false positive rates and was a key focus of our work. Our RFT approach shows significant promise here. By rewarding the model’s entire reasoning process, RFT encourages a more holistic understanding of the glove’s state, allowing it to learn the contextual difference between a true defect and a benign deformation artifact. This improved robustness against visual ambiguity represents a key advantage of our reasoning-driven methodology.

Addressing these limitations points toward critical avenues for future work. The primary focus will be on enhancing inference speed through techniques like model distillation and quantization to broaden applicability to real-time scenarios. Additionally, we plan to expand the dataset with more examples of challenging occlusions and further refine RFT strategies to improve overall detection accuracy and robustness.

In conclusion, this research serves as a strong proof-of-concept that MLLMs are poised to revolutionize automated anomaly detection. By shifting the paradigm from rigid, task-specific models to flexible, reasoning-driven systems, this work paves the way for more scalable, cost-effective, and intelligent quality control solutions in modern industry.
